# Mapping memory at scale: systems-level insights with translational and transdiagnostic relevance to fear-related disorders

**DOI:** 10.3389/fnbeh.2026.1844305

**Published:** 2026-07-01

**Authors:** Rodrigo O. Sierra, Sandra Ortega-Ferreira, Johanna M. Duran, Lizeth K. Pedraza

**Affiliations:** 1 Programa de Psicología, Facultad de Humanidades y Ciencias Sociales, Universidad Ean, Bogotá, Colombia; 2 Facultad de Ciencias Sociales y Humanas, Fundación Universitaria del Área Andina, Bogotá, Colombia; 3 Laboratorio de Bases Biológicas del Comportamiento, Facultad de Ciencias del Comportamiento, Universidad de La Sabana, Chía, Colombia

**Keywords:** engram, fear memory, network neuroscience, precision psychiatry, RDoC, systems consolidation, transdiagnostic

## Abstract

Fear-related disorders—including post-traumatic stress disorder (PTSD), specific phobias, and generalized anxiety disorder—are among the most prevalent and treatment-resistant psychiatric conditions. Despite decades of molecular and cellular advances, systemic pharmacological interventions and approaches targeting isolated brain regions have had limited therapeutic capacity, particularly in refractory patients. This mini-review argues that the translational impasse stems from a reductionist framework that treats mental disorders in general, and fear-related disorders in particular, as a localized phenomenon rather than a distributed network property. We synthesize recent evidence from engram research, oscillatory dynamics, and systems consolidation demonstrating that fear memories are encoded, maintained, and retrieved through brain-wide networks. We outline how emerging technologies such as high-density electrophysiology, calcium imaging, and functional neuroimaging combined with graph-theoretical analysis can map memory networks at scale, enabling precision interventions such as closed-loop neuromodulation and connectivity-based treatment prediction. Adopting a transdiagnostic perspective aligned with the Research Domain Criteria (RDoC), we position persistent aversive memory as a dimensional mechanism that crosses diagnostic boundaries, a perspective we propose as necessary for the development of more effective clinical interventions.

## 1 Introduction

Fear and anxiety-related disorders represent the most prevalent class of psychiatric conditions worldwide, affecting approximately 30% of the population across the lifespan (Kessler et al., 2005). Despite the substantial individual and societal burden, treatment resistance remains common: approximately 40%–60% of patients fail to achieve remission with first-line psychotherapy or pharmacotherapy (Craske et al., 2017).

The past three decades have yielded remarkable advances in understanding the molecular and cellular mechanisms of fear learning (LeDoux, 2000; Maren, 2001; Fanselow and Poulos, 2005). These advances in understanding fear acquisition and consolidation have been complemented by discoveries regarding memory disruption via protein synthesis-dependent reconsolidation (Nader et al., 2000), pharmacological and behavioral enhancement of fear extinction learning (Haubrich et al., 2017), and local electrical modulation approaches— including non-invasive neuromodulation targeting reconsolidation (Duran et al., 2022). Although these mechanisms have been characterized with increasing precision primarily in rodent models, the translation of these mechanistic insights into effective clinical interventions remains a persistent challenge. For example, pharmacological augmentation strategies—including propranolol for reconsolidation blockade and D-cycloserine for extinction enhancement—have produced inconsistent results in clinical trials, despite promising findings in animal models (Singewald et al., 2015).

We propose that this translational gap is not solely technical but conceptual. The dominant framework in fear memory research has privileged isolated brain structures such as the amygdala, hippocampus, and medial prefrontal cortex—as both the sources of potential psychopathologies and the targets of intervention. Although reductionist approaches have been essential for identifying molecular and cellular mechanisms with high precision, they inherently constrain the ability to capture how distributed circuits interact during memory processing. Moreover, many experimental techniques available in animal models—such as optogenetics and chemogenetics—cannot be directly applied in human clinical settings, further limiting the translational reach of single-region findings. However, converging evidence from memory engram research, systems consolidation, and network neuroscience reveals that fear memories are fundamentally distributed: they emerge from, and are maintained by, dynamic interactions across brain-wide circuits (Josselyn and Tonegawa, 2020; Haubrich et al., 2025a; Wen et al., 2024; Girardeau et al., 2017).

This mini-review advances three interconnected arguments. First, fear memory is a network-level phenomenon that cannot be adequately understood, or therapeutically modified, by targeting individual nodes. Second, emerging systems-level technologies provide unprecedented opportunities to map and modulate fear memory networks with temporal and spatial precision. Third, persistent aversive memories constitute a transdiagnostic dimension that cuts across categorical diagnoses, consistent with the Research Domain Criteria (RDoC) framework (Insel et al., 2010).

## 2 Neural networks and spatiotemporal encoding of fear memories

The search for the physical substrate of memory—the engram—has occupied neuroscience for more than a century. Early lesion studies, most notably Lashley’s (1950), who performed systematic cortical ablations of varying sizes and locations in rats and assessed their subsequent ability to learn and retain maze tasks, concluded that no single cortical region was essential for these memories. These findings led Lashley to formulate the principles of mass action and equipotentiality, positing that learning depends on the total amount of cortical tissue rather than its specific location. Although subsequent work has refined this interpretation, neither ablation nor removal of extensive neocortical regions selectively erased specific memory traces (Lashley, 1950), providing early evidence of the non-locality of memory encoding and consolidation.

**FIGURE 1:**
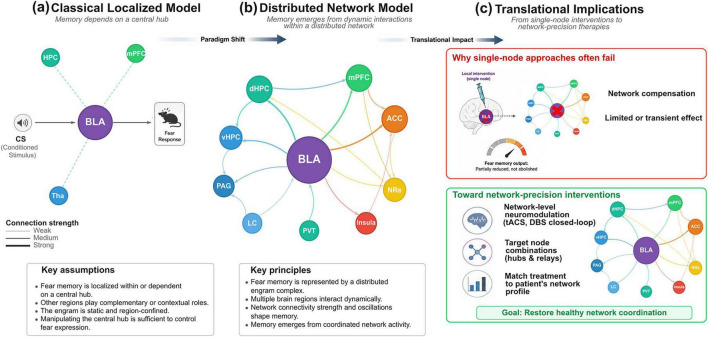
From localized to distributed fear memory models: conceptual framework and translational implications. **(a)** Classical localized model. Fear memory is conceptualized as dependent on a central hub that receives sensory information and drives behavioral output. Other regions are assigned complementary roles. Under this framework, the engram is treated as static and region-confined, and manipulating the central node is considered sufficient to control fear expression. **(b)** Distributed network model. Converging evidence from engram research, systems consolidation, and network neuroscience reveals that fear memory emerges from dynamic interactions within a distributed network of interconnected brain regions. Multiple nodes participate with varying connection strengths, forming a brain-wide engram complex. Memory is encoded, maintained, and retrieved through coordinated network activity, with connectivity strength and oscillatory coupling shaping memory expression. **(c)** Translational implications. Upper panel: Single-node interventions — such as pharmacological disruption of an individual hub — often produce limited or transient effects because the remaining network compensates through alternative retrieval routes and homeostatic plasticity. Lower panel: Network-precision interventions aim to restore healthy network coordination through three complementary strategies: (i) network-level neuromodulation targeting inter-regional oscillatory synchrony (e.g., tACS, closed-loop DBS); (ii) targeting node combinations — including critical hubs and relay structures — rather than isolated regions; and (iii) matching treatment to each patient’s individual network profile, informed by connectivity-based biomarkers. BLA, basolateral amygdala; dHPC, dorsal hippocampus; vHPC, ventral hippocampus; mPFC, medial prefrontal cortex; ACC, anterior cingulate cortex; NRe, nucleus reuniens; Tha/PVT, paraventricular thalamus; LC, locus coeruleus; PAG, periaqueductal gray; CS, conditioned stimulus; DBS, deep brain stimulation; tACS, transcranial alternating current stimulation.

Decades later, molecular neuroscience appeared to supply a more precise answer. The development of viral vector–based strategies and transgenic animal models harnessing the activity-dependent expression of immediate early genes (IEGs)—such as c-Fos and Arc promoter-driven systems—provided genetic access to neurons selectively recruited during specific experiences, thereby enabling their identification and causal manipulation. Combining these activity-dependent tagging approaches with optogenetics (light-activated ion channels enabling precise temporal control of neuronal activity) or chemogenetics (Designer Receptors Exclusively Activated by Designer Drugs, DREADDs, which enable sustained modulation of tagged populations over extended timescales) demonstrated that reactivating discrete populations, such as those in the basolateral amygdala or dorsal hippocampus, is sufficient to evoke learned behavior, whereas silencing them selectively impairs retrieval (Han et al., 2009; Kitamura et al., 2017; Liu et al., 2012). The identification of neuronal circuits in specific regions further allowed testing the hypothesis of selective elimination of memory traces (Han et al., 2009; Liu et al., 2014). These findings initially established the notion of a “static engram”: a discrete neural population whose activation reproduces the behavior and whose suppression abolishes it (Josselyn and Tonegawa, 2020). This earlier framework treated memory as localizable, intervenable, and discretely storable (Figure 1a). However, subsequent studies have revealed that silencing specific engram populations does not simply erase memory but can lead to compensatory redistribution of memory-related activity across neural networks (Tanaka et al., 2014; Haubrich and Nader, 2023).

However, this framework faces increasing empirical constraints. Kitamura et al. (2017) demonstrated that dorsal hippocampal neurons tagged during contextual fear conditioning in rodents become functionally silent by day 12, whereas medial prefrontal cortex neurons tagged in the same window undergo structural maturation and acquire the capacity to sustain remote memory. This suggests that circuits responsible for memory encoding and storage are subject to functional migration and dynamic restructuring over time (Guskjolen and Cembrowski, 2023; Tonegawa et al., 2018).

These findings are supported by evidence suggesting that memory engrams are continuously reconfigured. For instance, groups of neurons are constantly added or removed into dentate gyrus engrams as consolidation proceeds, and this turnover—not stable ensemble activity—predicts the emergence of memory specificity, referring to the progressive refinement of which stimuli a given engram responds (Tomé et al., 2024; Frankland et al., 2004; Guzowski et al., 2005). Brain-wide mapping of engram-labeled neurons has revealed that a single memory engages distributed populations across multiple cortical and subcortical areas, rather than being confined to canonical structures. Critically, simultaneous chemogenetic reactivation of multiple engram ensembles across regions produced stronger memory recall than reactivation of any single ensemble, supporting the “unified engram complex” hypothesis—that memory is stored and retrieved through coordinated interactions among distributed ensembles rather than through isolated nodes (Roy et al., 2022). The strength of these distributed representations predicts behavioral performance, with network-level changes emerging as the critical determinant of fear memory expression (Haubrich and Nader, 2023). These network dynamics are not static: Multiple neuromodulatory systems—including noradrenergic, dopaminergic, and cholinergic pathways—shape the functional architecture of memory networks by modulating encoding strength, consolidation dynamics, and the balance between memory specificity and generalization. As a well-characterized example, noradrenergic neuromodulation can redefine the cortico-subcortical networks that determine behavioral memory renewal, demonstrating that the functional architecture is malleable during causal manipulations (Haubrich et al., 2025b).

This shift from individual neurons to patterns of connectivity between them is further supported by Haubrich et al. (2025a), where, using fMRI in rodents during chemogenetic reactivation of engram-tagged neurons, it was observed that engram reactivation does not alter local activity in isolation but reconfigures the functional topology of the whole brain. Hubs of high centrality emerge in dorsal hippocampus, thalamic nuclei, and posterior parietal cortex, while other regions lose influence. Dynamics of coordination between structures is also supported by precise oscillatory mechanisms; specifically, theta/gamma coupling between hippocampus and medial prefrontal cortex during spatial memory consolidation predicts the efficacy of remote recall (Park et al., 2021), whereas experimentally induced desynchronization of these oscillations impairs fear memory (Xia et al., 2017). The basolateral amygdala modulates the power of these bands in prefrontal cortex during aversive memory consolidation, highlighting the role of interregional synchrony (Likhtik et al., 2014; Wen et al., 2022). Hippocampal sharp-wave ripples—brief high-frequency oscillatory events that coordinate reactivation of neural ensembles during sleep and quiet wakefulness—have been identified as a key mechanism driving systems consolidation, as their selective disruption impairs memory retention (Wilson and McNaughton, 1994; Girardeau et al., 2009). These ripples propagate coordinated activity patterns to widespread neocortical regions, providing a temporal framework for the redistribution of memory representations across brain-wide networks (Karimi Abadchi et al., 2020).

Systems consolidation is therefore not a linear transfer of information, but a reorganization of distributed brain networks supporting both recent and remote memory retrieval (Frankland and Bontempi, 2005). Recent computational and systems-level work further suggests that consolidation may follow structured trajectories within a high-dimensional space of distributed activity patterns, rather than a simple redistribution across regions (Sierra et al., 2026). Importantly, recent and remote memories engage distinct patterns of network activity and rely on different hub regions: while recent memories depend heavily on hippocampal-amygdalar circuits, remote memories increasingly recruit prefrontal, anterior cingulate, and thalamic nodes, reflecting a progressive shift in the spatial distribution of network hubs over time (Kitamura et al., 2017; Wheeler et al., 2013). Two prominent theoretical accounts frame this process differently. The Standard Consolidation Model (SCM) posits that memories initially depend on hippocampal-neocortical interactions but gradually become independent of the hippocampus as neocortical connections are strengthened over time (Frankland and Bontempi, 2005). In contrast, the Multiple Trace Theory (MTT) argues that the hippocampus remains essential for vivid, detailed recall regardless of memory age, with each retrieval episode creating new hippocampal traces (Moscovitch et al., 2016). Although these models differ in their predictions regarding hippocampal involvement in remote memory, both agree that memory is sustained by large-scale, distributed networks.

Considering the local and distributed dynamics of extensive brain regions participating in memory processing, trace persistence, rather than being sustained by the activation of the same neuronal ensemble, depends on reinstating patterns of functional connectivity that, although sustained by shifting populations, preserves a recognizable relational structure (Tanaka et al., 2014).

Ko et al. (2025) demonstrated that the passage of time rewires hippocampal engram circuits, enabling engram neurons to become rapidly active and guide behavior in related but non-matching situations—a process dependent on adult neurogenesis and sufficient to explain the emergence of generalized, gist-like memories. In parallel, Golbabaei et al. (2026) showed that prelimbic cortex sub-engrams with distinct projection profiles are differentially recruited over time, with basal amygdala- and lateral entorhinal cortex-projecting neurons supporting recent memory, while nucleus reuniens-projecting neurons are additionally recruited for remote recall. This aligns with the role of the nucleus reuniens as a critical relay in prefrontal-hippocampal communication during memory consolidation (Sierra et al., 2017). Notably, recent work has demonstrated that the nucleus reuniens is differentially engaged in remote versus recent memory processing: its projections to the basolateral amygdala are selectively recruited during remote fear extinction (Silva et al., 2021), while additional thalamo-septal pathways participate in the extinction of consolidated fear memories (Tomaszewski et al., 2026), highlighting the nucleus reuniens as a key hub in the temporal reorganization of fear memory networks.

Together, these findings support the concept of a metastable engram: a brain-wide, dynamically reconfiguring network representation of memory comprising functionally connected ensembles distributed across multiple regions (Josselyn and Tonegawa, 2020; Wen et al., 2024). Does ignoring large-scale dynamics compromise translational efforts in neuroscience research regarding persistent fear memories? The evidence suggests the answer is unequivocally yes. Far from affecting only basic neurobiology of memory research, findings on the distributed code sustaining engrams may be signaling that we are searching for local therapeutic targets for what is fundamentally a systemic phenomenon.

## 3 The translational gap: why promising lab findings fail in clinical settings

The reconsolidation hypothesis represents one of the most promising translational advances in fear memory research. Following the demonstration in rats that reactivated fear memories require *de novo* protein synthesis to restabilize memory traces (Nader et al., 2000), preclinical studies in rodents showed that pharmacological interventions during the reconsolidation window could permanently weaken conditioned fear responses (Tronson and Taylor, 2007). For instance, in rats, systemic or intra-amygdala administration of the β-adrenergic antagonist propranolol after memory reactivation selectively disrupted reconsolidation of auditory fear conditioning without affecting initial consolidation, suggesting that noradrenergic signaling is critical for the restabilization of reactivated fear traces (Debiec and Ledoux, 2004). Reconsolidation serves as a mechanism for memory updating, allowing incorporation of new information—including state-dependent contexts and appetitive content—into previously consolidated fear traces (Sierra et al., 2013; Haubrich et al., 2015). Similarly, extinction facilitated by the NMDA receptor partial agonist D-cycloserine and by retrieval-extinction paradigms (Monfils et al., 2009) showed robust effects in rodent models.

Translation to human clinical populations, however, has been inconsistent. Propranolol during memory reactivation produced encouraging results in healthy human volunteers (Kindt et al., 2009) but yielded mixed outcomes in PTSD patients across randomized controlled trials (Brunet et al., 2018). D-cycloserine augmentation of exposure therapy showed early promise, but larger trials revealed small and heterogeneous effects that did not replicate consistently across anxiety disorders (Hofmann et al., 2013; Smits et al., 2020). Preclinical evidence suggests that D-cycloserine efficacy depends critically on the integrity of cortical structures involved in inhibitory control (Sierra et al., 2016), and that dual-step pharmacological approaches combining reconsolidation-targeting agents may produce more robust effects (Soares et al., 2024).

Why have robust preclinical findings failed to translate? Based primarily on preclinical evidence, we argue that the answer lies in the network architecture of memory itself. The interventions share a common logic: they target a single molecular mechanism within a specific time window, assuming that memory depends on a localized, unitary process. Yet memories are sustained by distributed, redundant networks characterized by oscillatory synchrony across multiple nodes (Figure 1b). Evidence from rodent models suggests that when one node is pharmacologically disrupted, the network can compensate through homeostatic plasticity and alternative retrieval routes (Frankland and Bontempi, 2005; Tonegawa et al., 2018).

This redundancy explains several observations, including preservation of memory retrieval after hippocampal lesions (Lehmann et al., 2009) and persistence of hippocampal dependence over extended periods, consistent with multiple retrieval routes engaged depending on memory age and demand (Suzuki and Naya, 2011). Variable efficacy of reconsolidation-based interventions may reflect individual differences in network topology: more distributed fear memories may be less susceptible to single-node interventions. Resistance may also extend to aversive memories formed through intensive training, which in rats often renders them immune to standard attenuation mechanisms and specific molecular changes (Milekic and Alberini, 2002).

This distributed network perspective is further supported by studies demonstrating competition for behavioral control between fear expression and safety following extinction, where antagonistic processes coexist within specific regions—governed by transcriptional switches directing memory toward reconsolidation or extinction (de la Fuente et al., 2011) and competing patterns of theta-gamma coupling in the basolateral amygdala (Stujenske et al., 2014)—and across distributed networks through experience-dependent resonance in amygdalo-cortical circuits (Ozawa et al., 2020). Recent work has further demonstrated that memory retrieval engages distinct oscillatory dynamics within and across brain regions. Makino et al. (2024) showed that recall of recent fear memory is associated with enhanced slow gamma power (30–50 Hz) in the basolateral amygdala (BLA), whereas remote recall is characterized by stronger theta-phase (6–12 Hz) coupling from hippocampal CA1 and anterior cingulate cortex (ACC) to BLA gamma activity. Complementing this finding, Costa et al. (2025) demonstrated that, during retrieval of aversive scenes in humans, hippocampal gamma patterns that mirror amygdala encoding-related activity are reactivated, suggesting that emotional memory retrieval is supported by reinstatement of specific amygdala-driven hippocampal patterns. Extending this multi-regional perspective to subcortical circuits, Chen et al. (2025) identified that the anterior paraventricular thalamus (PVA) counteracts fear expression during retrieval by projecting to two parallel downstream pathways: the PVA–BLA circuit facilitates memory retrieval, while the PVA–ventral subiculum (vSub) circuit promotes active exploratory behaviors.

Critically, closed-loop brain stimulation coupled to hippocampal sharp-wave ripples—events coordinating synchronous activity across structures responsible for endogenous states conducive to the consolidation of new memories (Buzsaki, 2015) — has been shown to enhance fear extinction and reconfigure the emotional valence of aversive memories through precise stimulation of the medial forebrain bundle (Sierra et al., 2023). Together, these findings highlight that memory retrieval and consolidation rely on dynamic, multi-regional oscillatory interactions that vary with memory age, emotional content, and brain state.

## 4 Mapping memory at scale: tools for a systems-level approach

If fear memory is a network-level phenomenon, its investigation requires tools capable of capturing complementary aspects of distributed dynamics across multiple brain regions and spatial scales. Three technological developments are converging to enable this multi-scale approach.

High-density electrophysiology, exemplified by Neuropixels probes, enables simultaneous recording from thousands of neurons across multiple brain regions in behaving animals (Steinmetz et al., 2021; Jun et al., 2017). These probes span cortical and subcortical structures in a single penetration, enabling real-time measurement of coordinated activity patterns underlying fear memory encoding and retrieval. However, even with multiple simultaneous insertions, Neuropixels recordings sample neurons along discrete linear trajectories and thus capture only a small fraction of the total neuronal population, limiting their ability to provide a comprehensive whole-brain view (Siegle et al., 2021). Recent advances in large-scale, chronic recording platforms have further extended these capabilities: Melin et al. (2024) demonstrated simultaneous recordings from over 1,000 neurons across multiple brain areas in freely behaving mice, while the combination of μLED silicon probes with optogenetic perturbation enables both observation and causal manipulation of the same distributed circuits (Kinsky et al., 2023). Multisite electrophysiology approaches designed to study cross-regional communication during emotional behavior (Harris et al., 2017) are bringing the field closer to real-time mapping of distributed memory dynamics.

Miniaturized calcium imaging (miniscopes) complements electrophysiology by tracking the same neuronal populations across days to weeks, directly visualizing the engram dynamics—neuronal turnover, ensemble reorganization, and representational drift—that define systems consolidation (Ziv et al., 2013; Tomé et al., 2024). Studies combining miniscopes with fear conditioning show how hippocampal and amygdala engrams re-emerge after contextual fear relapse, providing evidence that distributed engram reactivation—rather than *de novo* encoding—underlies the return of fear (Zaki et al., 2022). Although miniscopes offer unparalleled longitudinal tracking of identified neuronal populations, their field of view is typically restricted to a single brain region, limiting the ability to simultaneously image distributed engram components.

Bridging the gap between cellular-resolution recordings in individual regions and whole-brain coverage, tissue-clearing methods such as iDISCO and CLARITY combined with light-sheet fluorescence microscopy enable brain-wide mapping of engram-labeled neurons at cellular resolution (Renier et al., 2016; Roy et al., 2022). These approaches have been instrumental in revealing the distributed nature of engram complexes, although they are inherently limited to post-mortem analyses and cannot capture the temporal dynamics of neural activity. At the human level, functional magnetic resonance imaging (fMRI) offers a complementary approach, providing the spatial coverage necessary to map brain-wide network states *in vivo*, albeit at substantially lower spatial and temporal resolution than cellular-level techniques. Graph-theoretical analyses identify critical hubs, quantify network efficiency, and detect community structure within fear circuits (Bullmore and Sporns, 2009; Bassett and Sporns, 2017). Dynamic functional connectivity analyses show that PTSD and anxiety disorders involve abnormal temporal fluctuations in prefrontal-amygdala coupling, rather than static hypo- or hyperconnectivity (Wen et al., 2022).

Integrating these approaches through computational modeling creates a multi-scale framework: from single-neuron engram dynamics (miniscopes), through mesoscale oscillatory coordination (Neuropixels), to whole-brain network topology (fMRI). This integration is essential because interventions at different spatial scales require corresponding biomarkers.

Electroencephalography (EEG) and multivariate pattern analysis have enabled identification of large-scale cortical electrical activity networks associated with memory acquisition, consolidation, and retrieval (Dai et al., 2017). The concept of “electome”—a mapping of brain electrical dynamics analogous to the connectome (Walder-Christensen et al., 2024; Mague et al., 2022)—offers a framework for characterizing network-level signatures of fear memory, with clinical potential through machine learning-based classification of EEG patterns (Williams, 2016). AI-assisted analysis of these electrophysiological signatures may provide predictive biomarkers accessible for clinical use, bridging laboratory-based network neuroscience and scalable diagnostics.

## 5 From distributed engrams to precision interventions

The systems-level framework here described opens concrete translational pathways. First, network-derived biomarkers—particularly oscillatory signatures and graph-theoretical measures of hub integrity—can serve as targets for closed-loop neuromodulation. Rather than delivering stimulation through fixed protocols, closed-loop systems detect maladaptive network states in real time and deliver precisely timed interventions, including deep brain stimulation (DBS), transcranial magnetic stimulation (TMS), or timed stimulus presentation (Ezzyat et al., 2018). This approach addresses network redundancy by targeting dynamic coordination between nodes rather than individual nodes. Transcranial alternating current stimulation (tACS) offers a non-invasive means of modulating inter-regional oscillatory synchrony with high temporal precision. Polania et al. (2012) demonstrated that simultaneous in-phase theta-band tACS applied to frontoparietal cortical sites enhanced cognitive performance, whereas anti-phase stimulation disrupted it, establishing a causal link between inter-regional phase synchronization and behavior. Extending this approach to fear memory networks—for example, by applying tACS to modulate prefrontal-amygdala theta coupling during extinction—could provide a clinically feasible method for reconfiguring maladaptive oscillatory coordination. Closed-loop stimulation may achieve effects that single-site interventions cannot. Multi-target neuromodulation delivering stimulation simultaneously or sequentially to multiple regions, further supports network-level interventions (De Ridder et al., 2015).

Second, individual differences in functional connectivity may predict treatment response. Drysdale et al. (2017) demonstrated that connectivity-based biotypes of depression predicted differential response to TMS, a finding with direct implications for anxiety disorders. Characterizing a patient’s fear memory network system before treatment may enable matching interventions—psychotherapy, pharmacological, or neuromodulation—to individual network profiles rather than diagnostic categories (Kas et al., 2025), advancing precision psychiatry in memory-based disorders (Figure 1c).

Third, wearable physiological monitoring — heart rate variability, electrodermal activity, actigraphy—provides ecologically valid indices of autonomic regulation associated with brain circuit function (Thayer et al., 2012; Sakaki et al., 2016). Integrating wearable data with neuroimaging-derived network models and dynamic psychometric measures, such as ecological momentary assessments (EMA), may enable continuous monitoring of treatment response and early detection of relapse risk, extending network-informed interventions beyond laboratory and clinic (Jacobson et al., 2020).

Although naturalistic biomarkers provide high ecological validity, controlled symptom provocation may offer complementary advantages. Zrenner et al. (2023) proposed that rapid switching between symptomatic and baseline psychiatric states during continuous EEG recording can isolate neurophysiological signatures reflecting the fluctuating circuit activity differentially engaged during symptom expression. This approach, analogous to classifier design in brain-computer interfaces, may enable extraction of circuit-specific EEG markers to guide personalized closed-loop interventions in fear and anxiety-related disorders.

## 6 Fear memory as a transdiagnostic dimension

The categorical framework of the DSM-5 (American Psychiatric Association, 2013), despite its clinical utility, assumes that anxiety disorders, PTSD, obsessive-compulsive disorder, major depression, and substance use disorders constitute discrete entities separable by symptom profiles (Hyman, 2010). This assumption is increasingly challenged. Comorbidity exceeds 55%, genetic susceptibility does not match the complex interaction of organisms with their environment, and individuals sharing the same diagnostic label exhibit striking heterogeneity in treatment response (Caspi and Moffitt, 2018).

The RDoC framework confronts these limitations by shifting attention from categorical diagnoses to dimensional constructs spanning units of analysis from genes to behavior (Insel et al., 2010). Within the negative valence domain, persistent aversive memory is not merely a symptom of PTSD; it is a core mechanism that traverses diagnostic boundaries (Merlo et al., 2024; Dalgleish et al., 2020).

If persistent aversive memory is transdiagnostic, interventions should target the mechanism rather than the diagnosis (Pedraza et al., 2022). The Unified Protocol (UP) for the Transdiagnostic Treatment of Emotional Disorders, exemplifies this approach by targeting shared maintaining processes, including emotional avoidance, aversive reactivity, and maladaptive emotion-driven behaviors, all converging on dysregulated fear and threat responses (Zarate-Guerrero et al., 2022; Farchione et al., 2012). A randomized controlled trial demonstrated that the UP significantly improved depressive and anxiety symptoms in patients with comorbid disorders, with effects maintained at 43 weeks (Ito et al., 2023). Similar efficacy has been reported in PTSD (Castro-Camacho et al., 2023), supporting its characterization as a memory-based disorder (Pedraza et al., 2022).

This convergence supports a central hypothesis: dysregulation of fear memory—across encoding, consolidation, retrieval, and failure of extinction—constitutes a shared network phenotype. This phenotype is not localized but emerges from distributed circuits encompassing amygdala, hippocampus, ventromedial prefrontal cortex, and insula (Lanters et al., 2024; Milad and Quirk, 2012). From a transdiagnostic perspective, comorbidity is not a nuisance variable to be statistically controlled, but an expected consequence of a shared mechanistic failure.

Research Domain Criteria framework, the Unified Protocol, and the emerging science of memory persistence converge on a key implication: the question is not which disorder a patient has, but which mechanisms are dysregulated. Persistent aversive memory is one such mechanism. However, physiological effects that these transdiagnostic interventions may have on the distributed neural networks underlying fear memory remain unexplored and constitute a promising frontier of translational science for future research.

## 7 Conclusion

This review argues that the persistent translational gap in fear and anxiety-related disorders reflects a mismatch between the distributed, dynamic nature of fear memory and the localized, static interventions that dominate preclinical and clinical practice. Three main conclusions emerge. First, fear memories are encoded, consolidated, and retrieved through brain-wide network states sustained by oscillatory synchrony, as demonstrated by recent findings showing that multi-regional oscillatory coordination—including theta-gamma coupling across amygdala, hippocampus, and prefrontal circuits—is dynamically modulated by memory age and emotional content. Second, emerging technologies—including high-density electrophysiology, calcium imaging, fMRI with graph-theoretical analysis, and EEG-based electome approaches— enable mapping of these networks at the resolution required for clinical translation, although important limitations remain: cellular-resolution approaches are largely restricted to post-mortem or single-region analyses in animal models, and non-invasive human techniques lack the spatial precision needed to resolve circuit-level mechanisms. Third, persistent aversive memories constitute a transdiagnostic mechanism that crosses categorical diagnostic boundaries, making this approach more accurate and realistic than diagnosis-guided treatments.

Future progress will require integrating molecular targets and critical brain regions while embracing the complexity of distributed memory systems. Closed-loop neuromodulation informed by network biomarkers, connectivity-based treatment matching, and continuous physiological monitoring represent concrete steps toward precision psychiatry of fear memory. Critical challenges for future research include: developing standardized multi-scale recording protocols that bridge preclinical and clinical paradigms; establishing the efficacy and safety of closed-loop neuromodulation targeting distributed network states in clinical populations; conducting longitudinal studies to track how fear memory network dynamics evolve across the lifespan and in response to treatment; and integrating transdiagnostic dimensional measures with network-derived biomarkers to enable truly personalized interventions. The question is no longer where fear memory resides, but how the network that sustains it can be reconfigured.

## References

[B1] American Psychiatric Association (2013). *Diagnostic and Statistical Manual of Mental Disorders*, 5th Edn. Washington, DC: American Psychiatric Association Publishing.

[B2] Bassett D. S. Sporns O. (2017). Network neuroscience. *Nat. Neurosci.* 20 353–364. 10.1038/nn.4502 28230844 PMC5485642

[B3] Brunet A. Saumier D. Liu A. Streiner D. L. Tremblay J. Pitman R. K. (2018). Reduction of PTSD symptoms with pre-reactivation propranolol therapy: A randomized controlled trial. *Am. J. Psychiatry* 175 427–433. 10.1176/appi.ajp.2017.17050481 29325446

[B4] Bullmore E. Sporns O. (2009). Complex brain networks: Graph theoretical analysis of structural and functional systems. *Nat. Rev. Neurosci.* 10 186–198. 10.1038/nrn2575 19190637

[B5] Buzsáki G. (2015). Hippocampal sharp wave-ripple: A cognitive biomarker for episodic memory and planning. *Hippocampus* 25 1073–1188. 10.1002/hipo.22488 26135716 PMC4648295

[B6] Caspi A. Moffitt T. E. (2018). All for one and one for all: Mental disorders in one dimension. *Am. J. Psychiatry* 175 831–844. 10.1176/appi.ajp.2018.17121383 29621902 PMC6120790

[B7] Castro-Camacho L. Barlow D. H. García N. Farchione T. J. Idrobo F. Rattner M. et al. (2023). Effects of a contextual adaptation of the unified protocol in multiple emotional disorders in individuals exposed to armed conflict in Colombia: A randomized clinical trial. *JAMA Psychiatry* 80 991–999. 10.1001/jamapsychiatry.2023.2392 37466983 PMC10357366

[B8] Chen J. Chen T. Zhu X. Dai X. Fang Z. Sun Y. et al. (2025). The anterior paraventricular thalamus counteracts fear expression during retrieval through both amygdala and subiculum circuits. *Commun. Biol.* 8:1635. 10.1038/s42003-025-09204-3 41272281 PMC12639042

[B9] Costa M. Pacheco-Estefan D. Gil-Nagel A. Toledano R. Imbach L. Sarnthein J. et al. (2025). Human hippocampal reactivation of amygdala encoding-related gamma patterns during aversive memory retrieval. *Nat. Commun.* 16:6820. 10.1038/s41467-025-61928-2 40707435 PMC12289934

[B10] Craske M. G. Stein M. B. Eley T. C. Milad M. R. Holmes A. Rapee R. M. et al. (2017). Anxiety disorders. *Nat. Rev. Dis. Primers* 3:17024. 10.1038/nrdp.2017.24 28470168 PMC11009418

[B11] Dai Z. de Souza J. Lim J. Ho P. M. Chen Y. Li J. et al. (2017). EEG cortical connectivity analysis of working memory reveals topological reorganization in theta and alpha bands. *Front. Hum. Neurosci.* 11:237. 10.3389/fnhum.2017.00237 28553215 PMC5427143

[B12] Dalgleish T. Black M. Johnston D. Bevan A. (2020). Transdiagnostic approaches to mental health problems: Current status and future directions. *J. Consult. Clin. Psychol.* 88 179–195. 10.1037/ccp0000482 32068421 PMC7027356

[B13] de la Fuente V. Freudenthal R. Romano A. (2011). Reconsolidation or extinction: Transcription factor switch in the determination of memory course after retrieval. *J. Neurosci.* 31 5562–5573. 10.1523/JNEUROSCI.6066-10.2011 21490196 PMC6622842

[B14] De Ridder D. Vanneste S. Langguth B. Llinas R. (2015). Thalamocortical dysrhythmia: A theoretical update in tinnitus. *Front. Neurol.* 6:124. 10.3389/fneur.2015.00124 26106362 PMC4460809

[B15] Debiec J. Ledoux J. E. (2004). Disruption of reconsolidation but not consolidation of auditory fear conditioning by noradrenergic blockade in the amygdala. *Neuroscience* 129 267–272. 10.1016/j.neuroscience.2004.08.018 15501585

[B16] Drysdale A. T. Grosenick L. Downar J. Dunlop K. Mansouri F. Meng Y. et al. (2017). Resting-state connectivity biomarkers define neurophysiological subtypes of depression. *Nat. Med.* 23 28–38. 10.1038/nm.4246 27918562 PMC5624035

[B17] Duran J. M. Sierra R. O. Corredor K. Cardenas F. P. (2022). Cathodal transcranial direct current stimulation on the prefrontal cortex applied after reactivation attenuates fear memories and prevent reinstatement after extinction. *J. Psychiatr. Res.* 145 213–221. 10.1016/j.jpsychires.2021.12.025 34929471

[B18] Ezzyat Y. Wanda P. A. Levy D. F. Kadel A. Aka A. Pedisich I. et al. (2018). Closed-loop stimulation of temporal cortex rescues functional networks and improves memory. *Nat. Commun.* 9:365. 10.1038/s41467-017-02753-0 29410414 PMC5802791

[B19] Fanselow M. S. Poulos A. M. (2005). The neuroscience of mammalian associative learning. *Annu. Rev. Psychol.* 56 207–234. 10.1146/annurev.psych.56.091103.070213 15709934

[B20] Farchione T. J. Fairholme C. P. Ellard K. K. Boisseau C. L. Thompson-Hollands J. Carl J. R. et al. (2012). Unified protocol for transdiagnostic treatment of emotional disorders: A randomized controlled trial. *Behav. Ther.* 43 666–678. 10.1016/j.beth.2012.01.001 22697453 PMC3383087

[B21] Frankland P. W. Bontempi B. (2005). The organization of recent and remote memories. *Nat. Rev. Neurosci.* 6 119–130. 10.1038/nrn1607 15685217

[B22] Frankland P. W. Bontempi B. Talton L. E. Kaczmarek L. Silva A. J. (2004). The involvement of the anterior cingulate cortex in remote contextual fear memory. *Science* 304 881–883. 10.1126/science.1094804 15131309

[B23] Girardeau G. Benchenane K. Wiener S. I. Buzsáki G. Zugaro M. B. (2009). Selective suppression of hippocampal ripples impairs spatial memory. *Nat. Neurosci.* 12 1222–1223. 10.1038/nn.2384 19749750

[B24] Girardeau G. Inema I. Buzsáki G. (2017). Reactivations of emotional memory in the hippocampus-amygdala system during sleep. *Nat. Neurosci.* 20 1634–1642. 10.1038/nn.4637 28892057

[B25] Golbabaei A. Josselyn S. A. Frankland P. W. (2026). PV-dependent reorganization of prelimbic cortex sub-engrams during systems consolidation. *Neuron* 114 142–158.e6. 10.1016/j.neuron.2025.09.033 41118757

[B26] Guskjolen A. Cembrowski M. S. (2023). Engram neurons: Encoding, consolidation, retrieval, and forgetting of memory. *Mol. Psychiatry* 28 3207–3219. 10.1038/s41380-023-02137-5 37369721 PMC10618102

[B27] Guzowski J. F. Timlin J. A. Roysam B. McNaughton B. L. Worley P. F. Barnes C. A. (2005). Mapping behaviorally relevant neural circuits with immediate-early gene expression. *Curr. Opin. Neurobiol.* 15 599–606. 10.1016/j.conb.2005.08.018 16150584

[B28] Han J. H. Kushner S. A. Yiu A. P. Hsiang H. L. Buch T. Waisman A. et al. (2009). Selective erasure of a fear memory. *Science* 323 1492–1496. 10.1126/science.1164139 19286560

[B29] Harris A. Z. Golder D. Likhtik E. (2017). Multisite Electrophysiology Recordings in Mice to Study Cross-Regional Communication During Anxiety. *Curr. Protoc. Neurosci.* 80 8.40.1–8.40.21. 10.1002/cpns.32 28678397 PMC5783183

[B30] Haubrich J. Nader K. (2023). Network-level changes in the brain underlie fear memory strength. *Elife* 12:R88172. 10.7554/eLife.88172 38047914 PMC10695559

[B31] Haubrich J. Crestani A. P. Cassini L. F. Santana F. Sierra R. O. Alvares Lde O. et al. (2015). Reconsolidation allows fear memory to be updated to a less aversive level through the incorporation of appetitive information. *Neuropsychopharmacology* 40 315–326. 10.1038/npp.2014.174 25027331 PMC4443944

[B32] Haubrich J. Machado A. Boos F. Z. Crestani A. P. Sierra R. O. Alvares L. O. et al. (2017). Enhancement of extinction memory by pharmacological and behavioral interventions targeted to its reactivation. *Sci. Rep.* 7:10960. 10.1038/s41598-017-11261-6 28887561 PMC5591313

[B33] Haubrich J. Russo G. Manahan-Vaughan D. (2025a). Sparse memory ensembles set brain-wide network states to sustain learned associations. *iScience* 28:113574. 10.1016/j.isci.2025.113574 41079610 PMC12514527

[B34] Haubrich J. Vera L. D. Manahan-Vaughan D. (2025b). Cortico-subcortical networks that determine behavioral memory renewal are redefined by noradrenergic neuromodulation. *Sci. Rep.* 15:9692. 10.1038/s41598-025-93263-3 40113948 PMC11926362

[B35] Hofmann S. G. Wu J. Q. Boettcher H. (2013). D-Cycloserine as an augmentation strategy for cognitive behavioral therapy of anxiety disorders. *Biol. Mood Anxiety Disord.* 3:11. 10.1186/2045-5380-3-11 23768232 PMC3686620

[B36] Hyman S. E. (2010). The diagnosis of mental disorders: The problem of reification. *Annu. Rev. Clin. Psychol.* 6 155–179. 10.1146/annurev.clinpsy.3.022806.091532 17716032

[B37] Insel T. Cuthbert B. Garvey M. Heinssen R. Pine D. S. Quinn K. et al. (2010). Research Domain Criteria (RDoC): Toward a new classification framework for research on mental disorders. *Am. J. Psychiatry* 167 748–751. 10.1176/appi.ajp.2010.09091379 20595427

[B38] Ito M. Horikoshi M. Kato N. Oe Y. Fujisato H. Yamaguchi K. et al. (2023). Efficacy of the unified protocol for transdiagnostic cognitive-behavioral treatment for depressive and anxiety disorders: A randomized controlled trial. *Psychol. Med.* 53 3009–3020. 10.1017/S0033291721005067 37449485 PMC10235654

[B39] Jacobson N. C. Summers B. Wilhelm S. (2020). Digital biomarkers of social anxiety severity: Digital phenotyping using passive smartphone sensors. *J. Med. Internet Res.* 22:e16875. 10.2196/16875 32348284 PMC7293055

[B40] Josselyn S. A. Tonegawa S. (2020). Memory engrams: Recalling the past and imagining the future. *Science* 367:eaaw4325. 10.1126/science.aaw4325 31896692 PMC7577560

[B41] Jun J. J. Steinmetz N. A. Siegle J. H. Denman D. J. Bauza M. Barbarits B. et al. (2017). Fully integrated silicon probes for high-density recording of neural activity. *Nature* 551 232–236. 10.1038/nature24636 29120427 PMC5955206

[B42] Karimi Abadchi J. Nazari-Ahangarkolaee M. Gattas S. Bermudez-Contreras E. Luczak A. McNaughton B. L. et al. (2020). Spatiotemporal patterns of neocortical activity around hippocampal sharp-wave ripples. *Elife* 9:e51972. 10.7554/eLife.51972 32167467 PMC7096182

[B43] Kas M. J. H. Penninx B. W. J. H. Knudsen G. M. Cuthbert B. Falkai P. Sachs G. S. et al. (2025). Precision psychiatry roadmap: Towards a biology-informed framework for mental disorders. *Mol. Psychiatry* 30 3846–3855. 10.1038/s41380-025-03070-5 40533548 PMC12240818

[B44] Kessler R. C. Berglund P. Demler O. Jin R. Merikangas K. R. Walters E. E. (2005). Lifetime prevalence and age-of-onset distributions of DSM-IV disorders in the national comorbidity survey replication. *Arch. Gen. Psychiatry* 62 593–602. 10.1001/archpsyc.62.6.593 15939837

[B45] Kindt M. Soeter M. Vervliet B. (2009). Beyond extinction: Erasing human fear responses and preventing the return of fear. *Nat. Neurosci.* 12 256–258. 10.1038/nn.2271 19219038

[B46] Kinsky N. R. Vöröslakos M. Lopez Ruiz J. R. Watkins de Jong L. Slager N. McKenzie S. et al. (2023). Simultaneous electrophysiology and optogenetic perturbation of the same neurons in chronically implanted animals using μLED silicon probes. *STAR Protoc.* 4:102570. 10.1016/j.xpro.2023.102570 37729059 PMC10510336

[B47] Kitamura T. Ogawa S. K. Roy D. S. Okuyama T. Morrissey M. D. Smith L. M. et al. (2017). Engrams and circuits crucial for systems consolidation of a memory. *Science* 356 73–78. 10.1126/science.aam6808 28386011 PMC5493329

[B48] Ko S. Y. Rong Y. Ramsaran A. I. Chen X. Rashid A. J. Mocle A. J. et al. (2025). Systems consolidation reorganizes hippocampal engram circuitry. *Nature* 643 735–743. 10.1038/s41586-025-08993-1 40369077

[B49] Lanters L. R. Öhlmann H. Langhorst J. Theysohn N. Engler H. Icenhour A. et al. (2024). Disease-specific alterations in central fear network engagement during acquisition and extinction of conditioned interoceptive fear in inflammatory bowel disease. *Mol. Psychiatry* 29 3527–3536. 10.1038/s41380-024-02612-7 38802508 PMC11541002

[B50] Lashley K. (1950). *In Search of the Engram. Symposium for the Society of Experimental Biology,# 4.* New York, NY: Cambridge University Press.

[B51] LeDoux J. E. (2000). Emotion circuits in the brain. *Annu. Rev. Neurosci.* 23 155–184. 10.1146/annurev.neuro.23.1.155 10845062

[B52] Lehmann H. Sparks F. T. Spanswick S. C. Hadikin C. McDonald R. J. Sutherland R. J. (2009). Making context memories independent of the hippocampus. *Learn Mem.* 16 417–420. 10.1101/lm.1385409 19553378 PMC2704104

[B53] Likhtik E. Stujenske J. M. Topiwala M. A. Harris A. Z. Gordon J. A. (2014). Prefrontal entrainment of amygdala activity signals safety in learned fear and innate anxiety. *Nat. Neurosci.* 17 106–113. 10.1038/nn.3582 24241397 PMC4035371

[B54] Liu X. Ramirez S. Pang P. T. Puryear C. B. Govindarajan A. Deisseroth K. et al. (2012). Optogenetic stimulation of a hippocampal engram activates fear memory recall. *Nature* 484 381–385. 10.1038/nature11028 22441246 PMC3331914

[B55] Liu X. Ramirez S. Redondo R. L. Tonegawa S. (2014). Identification and manipulation of memory engram cells. *Cold Spring Harb. Symp. Quant. Biol.* 79 59–65. 10.1101/sqb.2014.79.024901 25637263

[B56] Mague S. D. Talbot A. Blount C. Walder-Christensen K. K. Duffney L. J. Adamson E. et al. (2022). Brain-wide electrical dynamics encode individual appetitive social behavior. *Neuron* 110 1728–1741.e7. 10.1016/j.neuron.2022.02.016 35294900 PMC9126093

[B57] Makino Y. Wang Y. McHugh T. J. (2024). Multi-regional control of amygdalar dynamics reliably reflects fear memory age. *Nat. Commun.* 15:10283. 10.1038/s41467-024-54273-3 39653694 PMC11628566

[B58] Maren S. (2001). Neurobiology of Pavlovian fear conditioning. *Annu. Rev. Neurosci.* 24 897–931. 10.1146/annurev.neuro.24.1.897 11520922

[B59] Melin M. D. Khanal A. Vasquez M. Ryan M. B. Churchland A. K. Couto J. (2024). Large scale, simultaneous, chronic neural recordings from multiple brain areas. *bioRxiv [Preprint]* 10.1101/2023.12.22.572441 38187681 PMC10769364

[B60] Merlo S. A. Belluscio M. A. Pedreira M. E. Merlo E. (2024). Memory persistence: From fundamental mechanisms to translational opportunities. *Transl. Psychiatry* 14:98. 10.1038/s41398-024-02808-z 38355584 PMC10867010

[B61] Milad M. R. Quirk G. J. (2012). Fear extinction as a model for translational neuroscience: Ten years of progress. *Annu. Rev. Psychol.* 63 129–151. 10.1146/annurev.psych.121208.131631 22129456 PMC4942586

[B62] Milekic M. H. Alberini C. M. (2002). Temporally graded requirement for protein synthesis following memory reactivation. *Neuron* 36 521–525. 10.1016/s0896-6273(02)00976-5 12408853

[B63] Monfils M. H. Cowansage K. K. Klann E. LeDoux J. E. (2009). Extinction-reconsolidation boundaries: Key to persistent attenuation of fear memories. *Science* 324 951–955. 10.1126/science.1167975 19342552 PMC3625935

[B64] Moscovitch M. Cabeza R. Winocur G. Nadel L. (2016). Episodic memory and beyond: The hippocampus and neocortex in transformation. *Annu. Rev. Psychol.* 67 105–134. 10.1146/annurev-psych-113011-143733 26726963 PMC5060006

[B65] Nader K. Schafe G. E. Le Doux J. E. (2000). Fear memories require protein synthesis in the amygdala for reconsolidation after retrieval. *Nature* 406 722–726. 10.1038/35021052 10963596

[B66] Ozawa M. Davis P. Ni J. Maguire J. Papouin T. Reijmers L. (2020). Experience-dependent resonance in amygdalo-cortical circuits supports fear memory retrieval following extinction. *Nat. Commun.* 11:4358. 10.1038/s41467-020-18199-w 32868768 PMC7459312

[B67] Park A. J. Harris A. Z. Martyniuk K. M. Chang C. Y. Abbas A. I. Lowes D. C. et al. (2021). Reset of hippocampal-prefrontal circuitry facilitates learning. *Nature* 591 615–619. 10.1038/s41586-021-03272-1 33627872 PMC7990705

[B68] Pedraza L. K. Sierra R. O. de Oliveira Alvares L. (2022). Systems consolidation and fear memory generalisation as a potential target for trauma-related disorders. *World J. Biol. Psychiatry* 23 653–665. 10.1080/15622975.2022.2027010 35001808

[B69] Polanía R. Nitsche M. A. Korman C. Batsikadze G. Paulus W. (2012). The importance of timing in segregated theta phase-coupling for cognitive performance. *Curr. Biol.* 22 1314–1318. 10.1016/j.cub.2012.05.021 22683259

[B70] Renier N. Adams E. L. Kirst C. Wu Z. Azevedo R. Kohl J. et al. (2016). Mapping of brain activity by automated volume analysis of immediate early genes. *Cell* 165 1789–1802. 10.1016/j.cell.2016.05.007 27238021 PMC4912438

[B71] Roy D. S. Park Y. G. Kim M. E. Zhang Y. Ogawa S. K. DiNapoli N. et al. (2022). Brain-wide mapping reveals that engrams for a single memory are distributed across multiple brain regions. *Nat. Commun.* 13:1799. 10.1038/s41467-022-29384-4 35379803 PMC8980018

[B72] Sakaki M. Yoo H. J. Nga L. Lee T. H. Thayer J. F. Mather M. (2016). Heart rate variability is associated with amygdala functional connectivity with MPFC across younger and older adults. *Neuroimage* 139 44–52. 10.1016/j.neuroimage.2016.05.076 27261160 PMC5133191

[B73] Siegle J. H. Jia X. Durand S. Gale S. Bennett C. Graddis N. et al. (2021). Survey of spiking in the mouse visual system reveals functional hierarchy. *Nature* 592 86–92. 10.1038/s41586-020-03171-x 33473216 PMC10399640

[B74] Sierra R. O. Cassini L. F. Santana F. Crestani A. P. Duran J. M. Haubrich J. et al. (2013). Reconsolidation may incorporate state-dependency into previously consolidated memories. *Learn Mem.* 20 379–387. 10.1101/lm.030023.112 23782508

[B75] Sierra R. O. Nítola L. P. Duran J. M. Prieto D. R. León L. A. Cardenas F. P. (2016). Medial orbitofrontal cortex lesion prevents facilitatory effects of d-cycloserine during fear extinction. *Behav. Brain Res.* 296 379–383. 10.1016/j.bbr.2015.08.021 26306827

[B76] Sierra R. O. Pedraza L. K. Barcsai L. Pejin A. Li Q. Kozák G. et al. (2023). Closed-loop brain stimulation augments fear extinction in male rats. *Nat. Commun.* 14:3972. 10.1038/s41467-023-39546-7 37407557 PMC10322911

[B77] Sierra R. O. Pedraza L. K. Zanona Q. K. Santana F. Boos F. Z. Crestani A. P. et al. (2017). Reconsolidation-induced rescue of a remote fear memory blocked by an early cortical inhibition: Involvement of the anterior cingulate cortex and the mediation by the thalamic nucleus reuniens. *Hippocampus* 27 596–607. 10.1002/hipo.22715 28176459

[B78] Sierra R. O. Ramirez-Lugo L. Illescas-Huerta E. Bolanos A. R. Sotres-Bayon F. (2026). A selective cortico-limbic network organizes behavior during reward seeking under threat. *bioRxiv [Preprint]* 10.64898/2026.03.12.711336 PMC13445145 42559676

[B79] Silva B. A. Astori S. Burns A. M. Heiser H. van den Heuvel L. Santoni G. et al. (2021). A thalamo-amygdalar circuit underlying the extinction of remote fear memories. *Nat. Neurosci.* 24 964–974. 10.1038/s41593-021-00856-y 34017129

[B80] Singewald N. Schmuckermair C. Whittle N. Holmes A. Ressler K. J. (2015). Pharmacology of cognitive enhancers for exposure-based therapy of fear, anxiety and trauma-related disorders. *Pharmacol. Ther.* 149 150–190. 10.1016/j.pharmthera.2014.12.004 25550231 PMC4380664

[B81] Smits J. A. J. Pollack M. H. Rosenfield D. Otto M. W. Dowd S. Carpenter J. et al. (2020). Dose timing of D-Cycloserine to augment exposure therapy for social anxiety disorder: A randomized clinical trial. *JAMA Netw. Open* 3:e206777. 10.1001/jamanetworkopen.2020.6777 32496566 PMC7273198

[B82] Soares L. A. Nascimento L. M. M. Guimarães F. S. Gazarini L. Bertoglio L. J. (2024). Dual-step pharmacological intervention for traumatic-like memories: Implications from D-cycloserine and cannabidiol or clonidine in male and female rats. *Psychopharmacology* 241 1827–1840. 10.1007/s00213-024-06596-8 38691149

[B83] Steinmetz N. A. Aydin C. Lebedeva A. Okun M. Pachitariu M. Bauza M. et al. (2021). Neuropixels 2.0: A miniaturized high-density probe for stable, long-term brain recordings. *Science* 372:eabf4588. 10.1126/science.abf4588 33859006 PMC8244810

[B84] Stujenske J. M. Likhtik E. Topiwala M. A. Gordon J. A. (2014). Fear and safety engage competing patterns of theta-gamma coupling in the basolateral amygdala. *Neuron* 83 919–933. 10.1016/j.neuron.2014.07.026 25144877 PMC4141236

[B85] Suzuki W. Naya Y. (2011). Two routes for remembering the past. *Cell* 147 493–495. 10.1016/j.cell.2011.10.005 22036558

[B86] Tanaka K. Z. Pevzner A. Hamidi A. B. Nakazawa Y. Graham J. Wiltgen B. J. (2014). Cortical representations are reinstated by the hippocampus during memory retrieval. *Neuron* 84 347–354. 10.1016/j.neuron.2014.09.037 25308331

[B87] Thayer J. F. Ahs F. Fredrikson M. Sollers J. J. Wager T. D. A. (2012). meta-analysis of heart rate variability and neuroimaging studies: Implications for heart rate variability as a marker of stress and health. *Neurosci. Biobehav. Rev.* 36 747–756. 10.1016/j.neubiorev.2011.11.009 22178086

[B88] Tomaszewski K. F. Gajewska-Woźniak O. Ziółkowska M. Łukasiewicz K. Cały A. Sotoudeh N. et al. (2026). Projections from thalamic nucleus reuniens to medial septum enable extinction of remote fear memory. *Acta Neurobiol. Exp.* 86 30–52. 10.55782/zd27ss41 41885747

[B89] Tomé D. F. Zhang Y. Aida T. Mosto O. Lu Y. Chen M. et al. (2024). Dynamic and selective engrams emerge with memory consolidation. *Nat. Neurosci.* 27 561–572. 10.1038/s41593-023-01551-w 38243089 PMC10917686

[B90] Tonegawa S. Morrissey M. D. Kitamura T. (2018). The role of engram cells in the systems consolidation of memory. *Nat. Rev. Neurosci.* 19 485–498. 10.1038/s41583-018-0031-2 29970909

[B91] Tronson N. C. Taylor J. R. (2007). Molecular mechanisms of memory reconsolidation. *Nat. Rev. Neurosci.* 8 262–275. 10.1038/nrn2090 17342174

[B92] Walder-Christensen K. Abdelaal K. Klein H. Thomas G. E. Gallagher N. M. Talbot A. et al. (2024). Electome network factors: Capturing emotional brain networks related to health and disease. *Cell Rep. Methods* 4:100691. 10.1016/j.crmeth.2023.100691 38215761 PMC10832286

[B93] Wen Z. Pace-Schott E. F. Lazar S. W. Rosén J. Åhs F. Phelps E. A. et al. (2024). Distributed neural representations of conditioned threat in the human brain. *Nat. Commun.* 15:2231. 10.1038/s41467-024-46508-0 38472184 PMC10933283

[B94] Wen Z. Seo J. Pace-Schott E. F. Milad M. R. (2022). Abnormal dynamic functional connectivity during fear extinction learning in PTSD and anxiety disorders. *Mol. Psychiatry* 27 2216–2224. 10.1038/s41380-022-01462-5 35145227 PMC9126814

[B95] Wheeler A. L. Teixeira C. M. Wang A. H. Xiong X. Kovacevic N. Lerch J. P. et al. (2013). Identification of a functional connectome for long-term fear memory in mice. *PLoS Comput. Biol.* 9:e1002853. 10.1371/journal.pcbi.1002853 23300432 PMC3536620

[B96] Williams L. M. (2016). Precision psychiatry: A neural circuit taxonomy for depression and anxiety. *Lancet Psychiatry* 3 472–480. 10.1016/S2215-0366(15)00579-9 27150382 PMC4922884

[B97] Wilson M. A. McNaughton B. L. (1994). Reactivation of hippocampal ensemble memories during sleep. *Science* 265 676–679. 10.1126/science.8036517 8036517

[B98] Xia F. Richards B. A. Tran M. M. Josselyn S. A. Takehara-Nishiuchi K. Frankland P. W. (2017). Parvalbumin-positive interneurons mediate neocortical-hippocampal interactions that are necessary for memory consolidation. *Elife* 6:e27868. 10.7554/eLife.27868 28960176 PMC5655147

[B99] Zaki Y. Mau W. Cincotta C. Monasterio A. Odom E. Doucette E. et al. (2022). Hippocampus and amygdala fear memory engrams re-emerge after contextual fear relapse. *Neuropsychopharmacology* 47 1992–2001. 10.1038/s41386-022-01407-0 35941286 PMC9485238

[B100] Zarate-Guerrero S. Duran J. M. Naismith I. (2022). How a transdiagnostic approach can improve the treatment of emotional disorders: Insights from clinical psychology and neuroimaging. *Clin. Psychol. Psychother.* 29 895–905. 10.1002/cpp.2704 34984759

[B101] Ziv Y. Burns L. D. Cocker, Hamel E. O. Ghosh K. K. Kitch L. J. et al. (2013). Long-term dynamics of CA1 hippocampal place codes. *Nat. Neurosci.* 16 264–266. 10.1038/nn.3329 23396101 PMC3784308

[B102] Zrenner B. Zrenner C. Balderston N. Blumberger D. M. Kloiber S. Laposa J. M. et al. (2023). Toward personalized circuit-based closed-loop brain-interventions in psychiatry: Using symptom provocation to extract EEG-markers of brain circuit activity. *Front. Neural Circuits* 17:1208930. 10.3389/fncir.2023.1208930 37671039 PMC10475600

